# An Overview of *Aspergillus* Species Associated with Plant Diseases

**DOI:** 10.3390/pathogens13090813

**Published:** 2024-09-20

**Authors:** Latiffah Zakaria

**Affiliations:** School of Biological Sciences, Universiti Sains Malaysia (USM), Penang 11800, Malaysia; lfah@usm.my

**Keywords:** *Aspergillus*, crops, corn ear rot, cotton boll rot, peanut yellow mold, black mold, fruit rot, mycotoxins

## Abstract

The genus *Aspergillus* contains several species that are important plant pathogens. Plant pathogenic *Aspergillus* spp. affect agricultural crops in the field as well as after harvest, often associated with corn ear rot, cotton boll rot, peanut yellow mold, black mold of onion and garlic, fruit rot on grapes, pomegranates, olives, citrus, and apples. Coffee berries and coffee beans as well as tree nuts are also frequently infected by *Aspergillus* spp. Some of the plant pathogenic *Aspergillus* spp. are also mycotoxigenic, produced mycotoxin in the plant tissues leading to contamination of agricultural products. Over the years, reports of plant diseases caused by *Aspergillus* in various crops have increased, suggesting they are commonly encountered plant pathogens. This review focuses on agricultural crops or cultivated plants infected by *Aspergillus* spp. The compilation of plant pathogenic *Aspergillus* spp. provides information to mycologists, particularly those involved in plant pathology and crop protection, with updated information on plant diseases caused by various species of *Aspergillus*. The updated information also includes the locality or location, province, state and the country. The knowledge on the prevalence and geographic distribution of plant pathogenic *Aspergillus* spp. is beneficial in the application of crop protection.

## 1. Introduction

*Aspergillus* species are ubiquitous, found in various types of substrates, and distributed across all geographic areas and climatic conditions worldwide. Worldwide distribution of *Aspergillus* contributes to the conidia, which are common constituents of air, moving or drifting via air currents and spreading across both short and long distances. When conidia are deposited on a suitable substrate, they germinate when the conditions are suitable [1], colonizing the substrates via the degradation process. Agricultural crops and products, particularly food and feed, are common substrates of *Aspergillus*, leading to rotting or spoilage of crops and produce [1].

In earlier studies of plant pathogenic *Aspergillus*, *A. niger* and *A. flavus* have often been implicated in diseases of agricultural crops. Over the years, particularly after the introduction of the one fungus, one name concept, and taxonomic revision of the genus *Aspergillus* [2,3], other species have been reported as plant pathogens.

Black *Aspergillus* (section *Nigri*) often causes postharvest diseases in fruit crops, tree nuts, and vegetables, and is often found on peanuts, corn, onions, coffee, and grapes [4]. It is easy to recognize black *Aspergillus* as masses of black conidia appear on the infected parts of plants [5]. These conidia contain melanin in the cell wall, which protects them against UV light, drought, and high salt concentrations [6]. Species of black *Aspergillus* reported as plant pathogens are *A. niger*, *A. carbonarius*, *A. welwitschiae*, *A. ochraceus*, *A. awamori*, *A. aculeatus*, *A. tubingensis*, *A. japonicus*, *A. uvarum*, *A. foetidus*, *A. brasiliensis*, *A. aculeatinus*, and *A. sclerotiicarbonarius*, which are mentioned in this manuscript. Some of these species are producers of ocharatoxin such as *A. carbonarius*, *A. welwitschiae*, and *A. niger*. In addition, *A. niger* is also a fumonisin producer [7].

*Aspergillus* section *Flavi is* associated with plant diseases including *A. flavus*, *A. parasiticus*, *A. oryzae*, *A. tamarii*, and *A. minisclerotigenes.* Among the species, *A. flavus* is a well-known aflatoxin producer that is often associated with cottonseed, maize, peanuts, and tree nuts in the field and postharvest. *Aspergillus parasiticus* is also an aflatoxin producer particularly associated with peanuts.

Other species of *Aspergillus* that have been reported to be associated with plant diseases are *A. fumigatus* (section *Fumigati*), *A. westerdijkiae*, and *A. ostianus* (section *Circumdati*), *A. terreus* (section *Terrei*), *A. versicolor* (section *Versicolores*), *A. candidus* (section *Candidi*), *A. sulphureus* (section *Aspergillus*), and *A. ustus* (section *Usti*), as mentioned in this manuscript.

Due to taxonomic revision of the genus *Aspergillu*s, new species have been described [8,9] and may affect the identity of plant pathogenic species. As such, the information summarized in this work, including details on *Aspergillus* species associated with plant diseases, their occurrence, and geographic distribution, provides a valuable contribution that can assist professionals in this field in their efforts to address crop health and protection issues.

## 2. Pathogenicity of *Aspergillus* in Plants

*Aspergillus* species associated with plant diseases are generally opportunistic pathogens, and wounds or injuries are necessary for infection and colonization of the plant host [10]. Infection by *Aspergillus* usually occurs because of insect damage after drought or heat stress. For *Aspergillus* to cause disease, the conidia must germinate, followed by hyphal penetration and the colonization of the plant tissues. Subsequently, the plant host physiology is altered, and *Aspergillus* must adapt to the plant environment. After colonization and disease occurrence, conidia are produced and dispersed in the environment [10], and are an important factor for the survival of *Aspergillus* under hostile conditions [11].

The developmental stages of *Aspergillus* pathogenesis involve genes that enable infection and suppress resistance. The expression of the genes involved may be influenced by plant defense mechanisms and nutrient composition [12]. For *Aspergillus*, more data are available on pathogenesis in animals and humans than on pathogenesis in plants. However, according to Sexton and Howlett [10], fungal pathogenesis in animals, humans, and plants is similar, and information on pathogenesis in animals and humans can be applied to plants to understand disease mechanisms.

Conidial germination is an early stage of disease infection. Three morphological stages of conidial germination have been proposed: dormancy, isotropic growth, and polarized growth. Conidial dormancy is broken by several factors, including the presence of water and/or nutrients. Isotrophic growth is the swelling stage of conidia, which involves water uptake and the formation of new cell wall materials. The formation of a germ tube is known as polarized growth [11]. Ras protein and Cdc42/Rho GTPases are involved in fungal development and adaptation of fungal cells [13,14]. In polarized growth, *RasA* and *RasB* genes are essential in hyphal morphogenesis [13]. Detailed information on the genes and proteins involved in conidial germination and the formation of morphological stages of *Aspergillus* is provided in an earlier review by Baltussen et al. [11].

Germination of conidia leads to the formation of hyphae and mycelia, which subsequently enter and colonize the plant host. The conidia and hyphae are hydrophobic because they contain hydrophobins and globular proteins that are associated with pathogenicity, including hyphae attachment to plant tissues, the dispersal of conidia [15,16] and the increased longevity of aerial hyphae [17].

During the infection process, *Aspergillus* does not have access to the nutrients needed for its energy supply nor the biosynthesis of essential molecules to further colonize the plant tissues. To obtain the nutrients, the fungus depends on fatty acid metabolism, which is based on the glyoxylate cycle [18], which plays a role in fungal nutrition, and fungal virulence [19].

Lytic enzymes, such as proteases, are considered virulence factors in fungal pathogenesis as they are active in a wide pH range (pH 4–11) and have broad substrate specificity. *Aspergillus* also produces proteases for metabolism and pathogenicity [20,21]. Lytic enzymes are also useful for fungal colonization, nutrient uptake, adherence, and dissemination in plant tissues [22].

Melanin is a component of the fungal cell wall that confers resistance to UV light, protects against adverse environmental conditions, and contributes to fungal virulence [23]. Melanin also plays a role in conidial survival in plant hosts [10]. Furthermore, *Aspergillus* species have been reported to synthesize DHN melanin (1,8-dihydroxynaphthalene) and pyomelanin [24].

Superoxide dismutases (SOD) may also be virulence factors for the colonization of plant hosts by *Aspergillu*s, acting together with other virulence factors [25]. Several species, such as *A. niger*, *A. flavus*, *A. terreus*, and *A. nidulans*, have been reported to produce SOD [25]. Reverberi et al. [26] demonstrated that *A. flavus* exhibited transcriptional changes in both primary and secondary metabolism genes, depending on the substrate colonized, as a result of the trophic shift from saprobic growth to invasive pathogenic colonization. Pathogenic growth of the fungus in living kernels led to the upregulation of oxidative stress response pathway genes. Oxidative stress conditions arise at the fungus-host interface due to the plant’s defense mechanisms, and fungal pathogens have evolved strategies to detect and mitigate ROS accumulation, such as through the secretion of SOD and catalase, which convert ROS into less reactive molecules. Antioxidant mutants of *A. flavus* showed impaired growth and produced less aflatoxins, highlighting oxidative stress responses as a key factor in the switch from saprobic to pathogenic behavior.

In colonization of a plant host, mycotoxin is also a virulence factor that kills host tissues. For example, cyclopiazonic acid has been reported to be the main pathogenic factor in the colonization by *A. flavus* [27]. As for aflatoxin, Mehl et al. [28] suggested that the production of the mycotoxin in the soil gives the fungus a better competing ability against soil organisms, instead of functioning as a pathogenicity factor for colonization in plant tissues.

Colonization creates favorable conditions for the growth and development of *Aspergillus*. The fungus reproduces asexually within the plant tissues and produces conidia. Then, lesions form on the surface of the plant [10].

## 3. Common Plant Diseases Caused by *Aspergillus*

### 3.1. Corn Ear Rot

*Aspergillus flavus* is one of the main fungal species causing corn ear rot, although *A. parasiticus* and *A. niger* have also been reported to be associated with this disease [29]. Many studies on Aspergillus ear rot in corn-producing countries worldwide have focused on *A. flavus* as it is an aflatoxin producer [30,31,32,33].

*Aspergillus* species that cause corn ear rot survive in the soil and remain in crop debris, which becomes a source of inoculum. The infection of the ears occurs through silk during pollination and grain filling. Conidia from sources of inoculum land on the silk and germinate, develop in the silk, and grow downwards to colonize the ears [30]. The infection of the kernels occurs once the kernels are mature [31]. The growth of this pathogen is favored by high temperatures (>28 °C) and the high-water activity found in kernels. Under these conditions, *A. flavus* tends to become the predominant pathogen in corn kernels and develops during postharvest.

Drought stress and insect damage contribute to the susceptibility of corn plants to *Aspergillus* infection [29]. Drought and heat conditions lead to poor kernel development, which is suitable for the rapid growth of *A. flavus* as well as mycotoxin production [31]. Wound or injury produced by earworms and corn borers provide a point of entry for conidial infection. Drought stress intensifies insect damage to husks, which expedites the transmission of *A. flavus* [32,33].

The endophytic infection of corn ear rot by black *Aspergillus* may occur as the fungus has been isolated from healthy kernels. Moreover, some species of black *Aspergillus* can occur as biotrophic endophytes in corn [7]. Endophytic *A. flavus* has also been previously recovered from healthy corn [34,35].

The appearance of black or green conidial masses on the kernels is an indication of Aspergillus ear rot, which occurs at wound areas or near the ear tip. *Aspergillus flavus* typically form olive-green conidial masses, while *A. niger* forms a black coloration [29]. *Aspergillus* infection can occur in the field in maturing or mature kernels, as well as during harvest, storage, and processing. Infection in corn does not necessarily imply aflatoxin occurrence but clearly indicates an increased risk of contamination. For *A. flavus*, aflatoxin production occurs at a water activity of 0.87 [36] and an optimum temperature of 27–30 °C [37]. Under suitable conditions, aflatoxin can be produced within 24 h after infestation [38].

Ochratoxin A was also detected in corn under field conditions, suggesting an association between black aspergilli, especially *A. niger*, and corn during crop growth [7,39,40]. Thus, in addition to aflatoxin, ochratoxin is another *Aspergillus* mycotoxin that has the potential to contaminate corn.

### 3.2. Peanut Crown Rot, Root Rot, and Yellow Mold

Peanuts (*Arachis hypogaea*) are one of the most important cash crops cultivated worldwide for food and oil. The production of peanuts is affected by various fungal diseases, of which soil-borne diseases caused by *Aspergillus* species are among the most common diseases, leading to substantial losses. Soil-borne diseases in peanuts associated with *Aspergillus* include crown/collar rot, root rot, and yellow mold (Table 1).

#### 3.2.1. Crown/Collar Rot

Crown rot, also known as collar rot, is caused by *A. niger* and occurs in all peanut-producing countries. Economic losses due to crown rot are difficult to evaluate because the affected plants are scattered throughout the field; however, in some infected fields, losses of 50% have been reported [41]. According to Pande and Rao [42], the annual worldwide loss of peanut crops due to this disease is more than 10%.

The most common symptoms of crown rot are pre-emergence, post-emergence seedling damping-off, and sudden wilting. Young plants and seedlings are more susceptible than mature plants, which can lead to higher mortality rates. Older plants may become infected from the mid- to late season of planting [43].

Peanut seeds are susceptible to pathogens in moist soil environments. When the seeds germinate, the elongated shoots become infected, causing the hypocotyl to become water-soaked. Sudden wilting of the seedlings can be observed, as well as a rotation in the hypocotyl and cotyledon. Once infected, the hypocotyl and rotting roots are covered by black masses of conidia and mycelia. As infection occurs rapidly, peanut plants often die within 30 days, although others may survive longer [41,44].

*Aspergillus niger* causing crown rot in peanuts can be either soil-borne or seedborne. The pathogen is often present in the soil where peanuts are planted and can also often be found in the peanut seeds. This pathogen is prevalent in soils in which peanuts have been planted, often serving as the primary inoculum. The sporulation and growth of the pathogen mainly occurs under warm and moist conditions [41,45]. Outbreaks of crown rot are sporadic, with poor seed quality, changes in soil moisture due to high temperature during the seedling stage, drought stress, seedling damage due to pesticides, and feeding by roots and stem borers among the factors contributing to disease incidence [41].

#### 3.2.2. Yellow Mold

Yellow mold in peanuts is caused by yellow-green aspergilli, *A. flavus*, and *A. parasiticus*, which are saprophytes and facultative parasites in the soil, plant debris, rotting seeds, and peanut pods. These yellow-green aspergilli are also often found in healthy peanut pods. Both aflatoxigenic *A. flavus* and *A. parasiticus* infect and contaminate peanuts in the field. After harvest, during the drying and storage stages, aflatoxin is produced in the seeds, seedling stems, and pods. In the soil, *A. flavus* and *A parasiticus* occur as conidia and mycelia in plant debris and can infect the plant directly or when the plants are predisposed to several factors, such as damage by insects and nematodes, as well as dry weather [46].

During the preharvest infection and invasion of peanut seeds, *A. flavus* has been found to be more aggressive than *A. parasiticus* [46,47]. Excessive heat in the soil (27–30 °C) and lengthy drought periods (3–6 weeks) towards the end of the growing season favor *Aspergillus* invasion and aflatoxin production. During periods of drought, the leaf canopy recedes due to higher soil temperatures and soil moisture evaporation. These conditions disrupt the synthesis of phytoalexin, such that the growth of *Aspergillus* is no longer inhibited [46]. Severe drought causes permanent wilting, leaf shedding, and receding canopy, which leads to favorable conditions for the production of aflatoxin in peanut seeds [46].

The pre-emergence rotting of the seeds and seedlings are indicative of severe peanut infection. At this stage, necrotic lesions appear with sporulating *A. flavus* emerging on the hypocotyls, radicles, and cotyledons of both ungerminated and germinated seeds. This condition is known as yellow mold. When infected seedlings emerge, plant growth is stunted, the root system is poorly developed, and the leaves become chlorotic [46].

The contamination of peanuts with aflatoxin in the field increases during drought stress as the moisture in the seed is reduced, which can lead to pod damage caused by insects. These conditions also facilitate pathogen infection. Moreover, sucrose exudates from the roots and peanut pods contribute to the growth of *A. flavus* and *A. parasiticus* [48]. Insect damage in peanuts is favored by hot and dry conditions, and wounds on the pods encourage the penetration and colonization of pathogens. Infected seeds often display yellow-green discoloration, which may be associated with fungal sporulation. Seed infection may also occur without noticeable damage to the pod [46].

Aflatoxin contamination in peanuts is more prominent in tropical and subtropical regions [46]. Aflatoxins are commonly produced at moisture levels greater than 80% and temperatures exceeding 25 °C [49]. Inadequate drying favors fungal growth, and aflatoxins tend to accumulate in the plant seeds. Fungal growth can be controlled by drying peanut pods to 7% moisture and storing them at 25–27 °C at a relative humidity of 70% [50].

#### 3.2.3. Root Rot

*Aspergillus niger* has been reported to cause root rot in peanuts in the Laizi District, Shandong Province, China [51]. During infection, early symptoms in the peanut plants, including brown spots, appeared on the root and stem base, as well as the plants showing leaf chlorosis, stunted growth, and sudden wilting. Later, as the disease progressed, rot symptoms were also visible in the infected stem and root tissues, and numerous brown and black conidia were observed on the surface of the infected parts [51]. In this case, the causal pathogen was recovered from the infected roots and the stem base.

### 3.3. Cotton Boll Rot

One of the most serious diseases of cotton (*Gossypium herbaceum*) is boll rot, caused by a complex of fungal pathogens, of which *Aspergillus* species are among the pathogens. Boll rot was first reported in the late 1920s in the southwestern states of the USA. *Aspergillus niger* was recovered from several parts of infected cotton, including bolls, young dying squares or fellow buds, discolored pedicels, and lesions formed on the bracts [52]. Boll rot was initially known as smut, and the symptoms appeared only in injured bolls, mainly due to infestation by insects. During a survey on cotton disease in California in 1957–1960, several boll-rotting fungi, including *A. flavus* and *A. niger*, were found on cotton [53]. Table 2 shows the *Aspergillus* species associated with cotton boll rot reported in the USA and Bangladesh.

Cotton boll rot occurs in all cotton-producing countries and affects the yield and fiber quality of the resulting crop. Two species of *Aspergillus*, namely *A. niger* and *A. flavus*, are commonly associated with cotton boll rot [54]. However, most reports and publications have focused on *A. flavus*, possibly due to its aflatoxin contamination, which is the most notable problem related to the development of fibers and bolls. The contamination of cotton by aflatoxin has been reported in cotton-growing areas in the USA [55,56].

Most cotton boll pathogens, including *A. flavus*, are unable to penetrate healthy plant tissues. However, the conidia can enter the boll through wounds or holes made by aphids and other insects, including pink boll worms, tobacco budworms, boll weevils, and cotton stainers [57,58]. The infection of inner tissues affects the seeds and lint, which rot as a result. Dry and blackened bolls with black or brown spots are indicative of infection [54]. Temperature and humidity are the main parameters that influence *A. flavus* colonization, as well as the production of aflatoxin. Moist lint resulting from the opening of the boll is susceptible to infection, which causes the lint to weaken and results in the discoloration of the fiber [58].

### 3.4. Black Mold in Onion and Garlic

**Figure 1:**
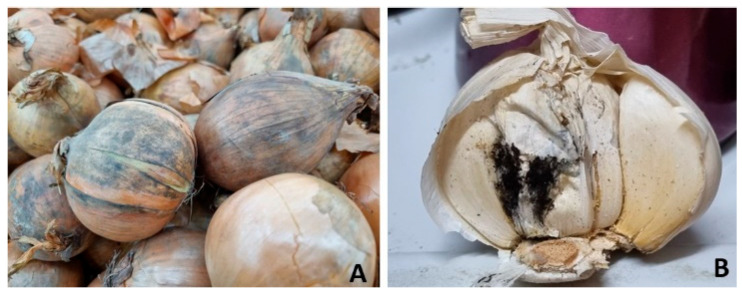
Black mold of onion (**A**) and garlic (**B**).

The infection of onions (*Allium cepa* L.) and garlic (*Allium sativum* L.) with black mold results in the appearance of black conidial masses on the bulbs (Figure 1A,B). On onions, conidial masses are formed between or on the outer layer of the scale leaves. Rot develops at the neck of the infected bulb, resulting in a shriveling of the scales. On garlic, dark brown or black conidial masses are formed on the bulb, and dry rot develops [59]. Black mold often occurs along the bulb veins, and a larger portion of the bulb is enveloped by conidia. The occurrence of black mold on onions and garlic gives the bulbs a sooty appearance.

Black *Aspergillus*, particularly *A. niger*, is often the causal pathogen of black mold in onion and garlic. Other *Aspergillus* species reported include *A. awamori* and *A. ochraceus* in garlic [60,61] and *A. welwitschiae* in onion [62,63,64] (Table 3).

The infection of onion and garlic bulbs by black mold can occur either in the field or during postharvest. *Aspergillus* species associated with black mold in onion and garlic are mainly saprophytes occupying plant debris and decaying organic matter and can turn into opportunistic pathogens by conidial infection. The conidia in the soil spread to the bulbs via the wind or rain. Conidia then enter the plants via wounds. Contaminated seeds are also sources of black mold inoculum [59]. In addition, endophytic *A. niger* has also been suggested as a vehicle of infection [7].

Black mold becomes apparent during storage, transportation, and sale. During the postharvest period, infection by black mold can cause significant losses, with bulbs becoming discolored and their tissues disintegrating. Black mold often occurs at high temperatures (27–30 °C) and humidity (70–80%), which can also lead to mycotoxin contamination [65]. As a result, the pathogens that cause black mold are widespread in hot and dry climates, but these can also be a problem in temperate areas when bulbs are stored at high temperatures and humidity levels. Moreover, the presence of black *Aspergillus* in onion seed samples has been reported to be prevalent in seeds grown or stored in warm climates.

Fumonisins (0.3 mg/kg) have been detected in onion samples from Hungary, albeit at low levels. In this case, the sample was contaminated with black *Aspergillu*s, identified as *A. awomori*, which was found in the fleshy part and outer layer of the onion bulb [66]. Fumonisin B2 has also been detected in onion samples in Taif, Egypt, wherein *A. welwitschiae* was identified as a potential fumonisin producer [62]. Ochratoxin has yet to be detected in onion and garlic bulbs. However, under suitable conditions, such as the optimum temperatures and humidity levels, there is always the possibility that black *Aspergillus* produces ochratoxin.

### 3.5. Aspergillus Fruit Rot

*Aspergillus* infects various types of fruit crops worldwide. Aspergillus rot is one of the main postharvest diseases affecting fruit crops and infected fruits cultivated in tropical, subtropical, and temperate regions. Among these, Mediterranean fruit crops are susceptible to Aspergillus rot. Infection often occurs during the harvest period, and the most common *Aspergillus* species associated with fruit crops is black *Aspergillu*s, especially *A. niger*, with other species including *A. flavus*, *A. fumigatus*, *A. tubingensis*, *A. parasitus*, *A. awamori*, *A. terreus*, *A.welwitschiae*, *A. uvarum*, and *A. japonicus* [67].

**Figure 2:**
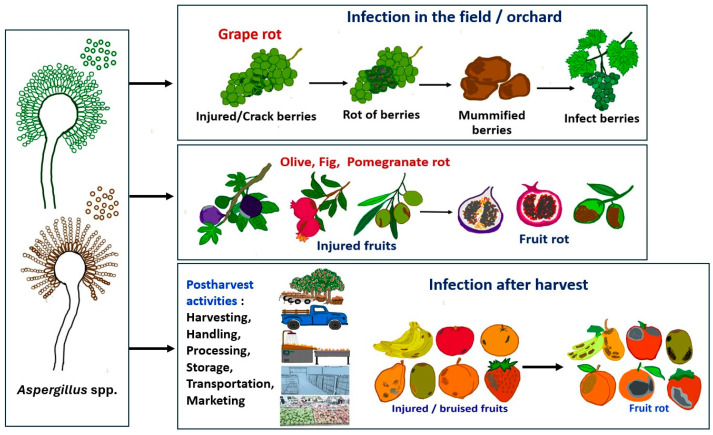
Infection of *Aspergillus* spp. on various fruit crops in the field and after harvest.

Figure 2 illustrates the infection of *Aspergillus* in fruits crops. The infection of fruit crops by *Aspergillus* occurs in the field, during harvest, and postharvest. In the field, when the sugar content increases during fruit maturation, the population of *Aspergillus* increases. When fruits are wounded, *Aspergillus* can easily infect these weakened fruits. *Aspergillus* also infects fruits during harvesting, handling, storage, washing, grading, packing, transportation, and sale, up until the product is bought by consumers [68]. Postharvest *Aspergillus* infection usually occurs via bruises, or other cuts on fruits, as well as through natural openings. Infection is favored by conditions of high temperatures and moisture, which promote conidial germination and fungal growth [68]. Moreover, wounds lead to the release of nutrients and water from the cells, providing suitable conditions for fungal growth. Postharvest fruit rot can lead to huge losses in storage and supply chains since fruits with rot symptoms are unmarketable and unsuitable for consumption.

Aflatoxin and ochratoxin A produced by mycotoxigenic *Aspergillus* have been detected in grapes, figs, pomegranates, and olives, as well as products based on these fruit crops. Studies on mycotoxin contamination of these fruit crops have received much more attention compared to studies on mycotoxins in tropical fruit crops, which remain scarce.

#### 3.5.1. Grapes Bunch Rot, Sour Rot, and Vine Canker

Grapes (*Vitis vinifera*) are one of the most important fruit crops in the world, and are mainly cultivated for wine production (71%). Only 27% of grapes are consumed fresh, while 2% are turned into dried fruits [69]. In vineyards, *Aspergillus* species infect grape berries, particularly during the summer when the conditions of high moisture and temperatures of 20–30 °C are prevalent [69,70]. During maturation, the rates of infection by *Aspergillus* spp. are higher, and black *Aspergillus* dominates at temperatures higher than 37 °C [71]. Occasionally, *A. flavus* and *A. parasiticus* have been isolated from grapes [72,73]. Some strains of pathogenic *Aspergillus* species are also mycotoxigenic, contaminating grapes, as well as their corresponding final products. In the postharvest period, grapes are processed according to their intended use. During these processes, contamination by *Aspergillus*, as well as other fungi, can occur [69].

Bunch rot, vine canker, and sour rot are diseases often associated with black *Aspergillus* in vineyards. The main sources of the inoculum of black *Aspergillus* in vineyards are soil and vine debris, from which wind-borne conidia are deposited onto the surface of the berries [74]. Black *Aspergillus*, which infects grape berries, is regarded as a secondary invader or opportunistic pathogen that causes infection when the berries are injured or wounded by insects or mechanical impact [75]. Prevalent black *Aspergillus* species found in infected grapes include *A. niger*, *A. carbonarius*, *A. aculateus*, *A. japonicus*, and *A. uvarum* [76,77], as well as occasionally *A. tubingensis* [77] and *A. awamori* [78]. These species are frequently reported to cause disease in grapes (Table 4).

Several black *Aspergillus* species are ochratoxin producers, and ochratoxin A is produced during veraison to ripening. Although *A. carbonarius* is the main producer of ochratoxin A, to a certain extent, *A. niger, A. tubingensis*, and *A. awamori* also contribute to ochratoxin A contamination in grape berries [79,80]. The contamination of ochratoxin A in wine was first reported by Zimmerli and Dick [80]. Subsequently, studies on ochratoxin A in wine and other grape products have increased [69,81,82,83,84,85].

**Table 4:** *Aspergillus* spp. associated with diseases of grape berries.

Grapes (*Vitis vinifera*)	*Aspergillus* spp.	Country	References
Disease			
Bunch rot	*A. aculeatus*	southwestern Ontario	[86]
	*A. niger*	Chile	[87]
	*A. carbonarius*	Victoria, Australia	[88]
	*A. niger*, *A. carbonarius*	-	[89]
	*A.tubingensis*	Kimcheon-si, Gyeongbuk province, Korea	[90]
		Gimcheon, South Korea	[77]
Sour rot	*A.carbonarius*	Kern County, California	[91]
	*A. niger*, *A. aculeatus*, *A. oryzae*	Yantai, Shandong Province, China	[92]
	*A. niger*, *A. carbonarius*	Rhodes, Greece	[93]
		Central and Southern Joaquin Valley, California	[94]
Vine canker	*A. niger*	San Joaquin Valley, California	[95]
	*A. niger*	southeastern Sicily, Italy	[96]
	*A. niger*, *A. tubingensis*, *A. carbonarius*	Sicily, Italy	[97]
	*A. niger* and/or *A. tubingensis*	Fresno and Sonoma counties, California	[98]

*Aspergillus niger* and *A. awamori* (now known as *A. welwitschiae*) are also fumonisin producers. Similar to ochratoxin A, fumonisin contamination has been reported in wine and other grape products [99,100,101,102,103]. According to Varga et al. [99], the accumulation of fumonisins can occur during the drying process, as mycotoxins are present before drying.

##### Bunch Rot

Bunch rot in grapes is caused by a range of fungi, including *Aspergillus*, which infect grape berries through wounds. Fungal pathogens that infect berries can sometimes be identified based on their conidial appearance. *Aspergillus* produces dark brown or black conidia, *Botrytis* produces gray conidia, and *Penicillium* produces green conidia [104]. Several *Aspergillus* species (Table 4) have been reported to be associated with grape bunch rot, including *A. niger*, *A. carbonarius* [87,88,89], *A. aculateus* [86], and *A tubingensis* [77,90].

Infection with bunch rot pathogens starts at the site of a wounded area and spreads rapidly to the entire grape cluster. Brown spots emerge on the berries, and as the disease progresses, the berries rot and black-to-dark brown conidia appear. Rotted berries become soft, shrivel, or collapse [104]. Bunch rot development is influenced by the wound on the berries and the compactness of the berry cluster. The sugar content increases as the fruits ripen, which increases the susceptibility of the wounded berries to infection by bunch rot pathogens [104]. Growth pressure on grape berry clusters leads to splitting or cracking. Bunch rot infection is favored by warm and wet conditions, with prolonged wet conditions leading to an increased rotting of berries [89,104]. Severe outbreaks of this disease can occur during periods of harvest under warm conditions [87].

Kazi et al. [88] studied the infection process of *A. carbonarius* in grape berries. Their findings showed that infection can occur at any stage of berry development if the inoculum is sufficient. Lower infection was found to occur when berries were small, green, and hard, which suggests that young berries are resistant to *A. carbonarius* infection. Infection was generally higher during veraison and harvest, which is similar to the findings reported by Battilani et al. [105] and Ponsone et al. [106]. Guzev et al. [107] also reported that infection was very low before veraison but often higher at harvest. The occurrence of black *Aspergillus* was also higher at harvest [105], which contributes to the incidence of bunch rot.

##### Sour Rot

Bunch rot often leads to sour rot, which causes the infected berries to appear wet due to leaking of juice or the oozing of the berry tissues, resulting in the cracking and collapse of the berries, which also enhances the growth of yeast and bacteria [104,108]. This disease is also known as summer bunch rot. Grape sour rot is a complex disease involving filamentous fungi, yeasts, acetic acid bacteria, and fruit flies. The disease is characterized by the smell of acetic acid or vinegar, as yeasts convert sugars to ethanol. Ethanol is then oxidized to acetic acid by the bacteria [109]. Fruit flies attracted to the sour smell act as vectors, spreading the filamentous fungi, yeasts, and acetic acid bacteria. Fruit flies may also cause injury to grape berries, which facilitates infection, particularly by fungi and bacteria [108,110]. Sour rot development is conducive to a high relative humidity and longer periods of wetness [94]. The main notable difference between bunch rot and sour rot is the vinegar-like smell caused by the accumulation of ethanol and acetic acid. Both diseases result in economic losses as they affect the berries, which in turn affects the final products.

Many filamentous fungi are involved in the sour rot of grapes, including *Aspergillus*, of which *A. niger* and *A. carbonarius* are frequently found on infected berries (Table 4). Both *A. niger* and *A. carbonarius* colonize wounded berries, causing bunch rot, followed by sour rot. *Aspergillus niger* and *A. carbonarius* have both been recovered from berries affected by sour grapes on the island of Rhodes, Greece [93]. Later, Rooney-Latham et al. [91] found that *A. carbonarius* was the main organism recovered from berries infected with sour rot in California. Findings by Gao et al. [92] indicated that *A. niger*, *A. aculeatus*, and *A. oryzae* are involved in sour rot in Yantai, Shandong Province, China.

##### Vine Canker

Grapevine canker is commonly associated with fungal pathogens in the families *Botryosphaeriaceae*, *Diatrypaceae*, and *Diaporthaceae*. Typical symptoms of grapevine canker include necrosis of the internal part of the trunk, indicating the formation of canker, the dieback of cordons or the whole vine, stunted shoot development, shoot death, rotting, and the dropping of berry clusters [98,111].

*Aspergillus* species causing vine canker have been reported in San Joaquin Valley, California, and southeastern Sicily, Italy. In California, Michailides et al. [95] reported *A. niger* as the causal pathogen of vine canker, in which the disease was detected in one-year-old cv. Redglobe vines. The disease was detected in the crotch, branching, and along shoots. Abundant black conidia were observed within the canker, as well as on the surface of the canker. Vitale et al. [97] identified *A. niger*, *A. tubingensis*, and *A. carbonarius* as pathogens of vine canker in Italy, of which the virulence was equal among the three species. Most canker lesions were detected at branch points and on the stems of young shoots, of which the infected tissues were discolored, and some were dead. Black powdery conidia are abundant and sometimes appear on the surface of lesions [96]. A recent study by Zhuang et al. [98] on vine canker in California indicated that *A. niger* and *A. tubingensis*, or both may be the causal pathogens of this disease (Table 4). Further studies on the species confirmations are currently underway.

Infection by *Aspergillus* causing vine canker occurs through wounds due to the removal of lateral shoots or leaves, particularly when the vine is topped to form cordons. Another method involves growth cracks that often occur in fast-growing one-year-old shoots [95,97]. *Aspergillus* causing vine canker usually forms black sporulations on the surface and underneath the affected bark tissues, which is the main characteristic differentiating this infection from other vine canker fungal pathogens. Moreover, multiple canker lesions appear on the cordon, spurs, and trunk of the vine, with visible brown discoloration in the xylem tissues. Infected tissues also typically show sporulation, necrosis, and black discoloration [98].

### 3.6. Fig Fruit Rot

Fig (*Ficus carica*) is mainly cultivated in the Mediterranean regions as the plant is well adapted to the Mediterranean climate, with its hot and dry summers and cold winters. Although fig is widely cultivated in this region, it can also be cultivated in humid tropical and subtropical regions [112]. For commercial purposes, fig fruits are converted into dried or preserved forms. Fig fruits are sold fresh for local consumption, as the fruits are easily perishable, and their shelf life is short [113]. Because the skin of the fruit is soft, it is easily wounded or damaged, and is thus susceptible to infection by fungi. Moreover, owing to their high sugar content, various fungi can grow on these fruits, which can lead to fruit rot [114].

Aspergillus fig rot is caused by several species, including *A. flavus*, *A. parasiticus*, *A. fumigatus*, *A. niger*, *A. japonicus*, and *A. carbonarius* [115,116,117], as shown in Table 5. *Aspergillus* causes rot in fresh fig fruits and smut in dried figs. Fig cultivars with larger ostioles are more susceptible, as the ostiole is a natural opening, which permits the fungi to enter the internal tissues of the fruit. When fruits ripen, abundant conidial masses are formed in the infected tissues [117]. Wounded or damaged fruits are also susceptible to *Aspergillus* infection, as the fungi can directly infect fruits.

Ripe and sun-dried fig fruits are susceptible to *Aspergillus* infection, which provides favorable conditions for mycotoxin production [118]. According to Buchanan et al. [118] and Iamanaka et al. [120], dried figs are susceptible to infection by *A. flavus* and *A. parasiticus.* Both species have often been recovered from dried figs [115,119]. Based on a study by Heperkan and Karbancioglu-Güler [121], *A. flavus* was found to be prevalent in dried fig, while *A. parasiticus* was not frequently isolated. Due to the presence of *A. flavus* and *A. parasiticus*, aflatoxins were detected, particularly in dried figs [121,123,124].

Mycotoxigenic black *Aspergillus*, particularly *A. niger* and *A. carbonarius*, have been isolated from diseased figs. *Aspergillus niger* and *A. carbonarius* are also prevalent during sun-drying, and both species are tolerant to ultraviolet rays, contributing to their prevalence during the drying process [71]. Similar to aflatoxins, ochratoxin A has been reported in dried figs [125,126,127,128,129].

Despite the susceptibility of fig to *Aspergillus* infection, as well as the contamination of fruit and fig products with aflatoxins and ochratoxin A, the level of contamination is generally low [118].

### 3.7. Olive Fruit Rot

*Aspergillus* is also the most common fungal flora recovered from olive fruits (*Olea europaea*) and has been isolated from fruit rot lesions, as well as from fruits infested by fruit flies [130,131]. According to Lazzizera et al. [132], most fungi associated with olive fruit rot, including *Aspergillus*, are secondary invaders or saprophytes, as the fungi infect olive fruits through wound, unlike *Colletotrichum* and *Botryosphaeriaceae* fungi, which directly infect olive fruits. In a study by Chliyeh et al. [133], *A. flavus* was found to only infect olive fruits through wounded fruit epicarps.

*Aspergillus* species that have been isolated from olive fruit rot (Table 6) include *A. ochraceus*, *A. fumigatus*, *A. flavus*, and *A. niger* [130,133,134,135]. *Aspergillus niger* and *A. tubingensis* were isolated from olive fruits infested with olive fruit flies [136]. In fresh olive fruits, *A. fumigatus*, *A. niger*, and *A. tubingensis* have also been reported [131,137], which may indicate that these species are endophytes. Endophytic *Aspergillus* species have been recovered from the twigs and roots of olive trees [137,138]. The main concern regarding *Aspergillus* growth on olive fruits is contamination by mycotoxigenic aspergilli, which can affect the production of olive oil.

As olives are stored after harvest, improper storage can promote the growth of mycotoxigenic *Aspergillus*, and the production of aflatoxin and ochratoxin A. The occurrence of mycotoxigenic fungi on olive fruits may lead to the contamination of olive oil with mycotoxin. In fact, the co-occurrence of aflatoxins and ochratoxin A has been reported in olive oil in southern Italy [139,140]. Ochratoxin A has also been reported in extra virgin oil [141] and in olive oil of Greek origin [142,143]. Aflatoxins have also been detected in olive oil in Greece [144], Iran [145], and Spain [146].

Although aflatoxins and ochratoxin A have been reported in olive fruits and olive oil, the level of contamination tends to be low, and it is believed to not affect consumers or cause any public health concerns. However, the continuous intake and exposure to contaminated products can pose a significant risk to consumers [140,147,148]. Moreover, the cumulative intake of these olive products may lead to health concerns.

### 3.8. Pomegranate Fruit Rot

Aspergillus rot of pomegranate (*Punica granatum*) is commonly associated with fruit rot and heart rot in pomegranates. This tends to start in the field during flowering and early fruit development, particularly after rainfall. Rot symptoms appear on the external part of the fruits near the calyx and manifest as discoloration of the rind, with the rind turning paler red or brownish red. Inside infected fruits, black powdery conidia are apparent, resulting in the rotting of the arils and the cracking of the fruit [149,150]. In a study by Ezra et al. [151], *Aspergillus* was found to cause fruit rot by penetrating the fruit through a damaged crown, resulting in the rotting of the fruit mesocarp tissue; however, rotting of the arils was not observed. Although pomegranate heart rot did not cause any noticeable symptoms on the rind, the arils rotted, and fungal mycelia were observed. As a result of the different stages of rot development, some arils exhibited brown/soft rot as well as black/dry rot [151].

In most cases, black aspergilli are associated with pomegranate fruit rot, which can occur both in the field and postharvest (Table 7). In an earlier study, *A. variecolor*, *A. awamori*, *A. fumigatus*, *A. flavus*, and *A. niger* were found to be causal pathogens of pomegranate fruit rot [152]. Pomegranate fruits in orchards near Cairo, Egypt, were found to be infected with *A. niger*, of which the fungus was isolated from the internal parts of the fruits. *Aspergillus niger* was also reported to cause the soft rot and dry rot of pomegranates in Shaanxi Province, China [153]. Infection by *A. niger* subsequently facilitates infection with bacteria and yeast [154].

After the revision of the taxonomy and nomenclature of the genus *Aspergillus*, other species have been found to be associated with pomegranate fruit rot during the preharvest and postharvest periods. In southern Italy, *A. tubingensis*, *A. welwitschiae*, *A. japonicus*, and *A. uvarum* are associated with postharvest pomegranate fruit rot [156]. According to Mincuzzi et al. [157], *A. tubingensis* and *A. welwitschiae* were the main species causing pomegranate fruit rot, whereas *A. uvarum* and *A. japonicus* were minor species. Preharvest pomegranate fruit rot in Greece and Cyprus were found to be mainly caused by *A. niger* and *A. tubingensis*, although various fungal pathogens, including *Alternaria*, *Colletotrichum*, and *Botrytis*, were also associated with fruit rot [158]. Guo et al. [155] reported *A. tubingensis* as a causal pathogen of pomegranate fruit rot in China.

Pomegranate fruit rot not only reduces the yield and quality of the fruits but also contaminated fresh and processed fruits with ochratoxin and fumonisin. Kanetis et al. [158] reported approximately 20% of *A. niger* isolates associated with pomegranate fruit rot could produce ochratoxin A in vitro. Isolates of *A. tubingensis* (33%) from Greece also produced ochratoxin A. Only *A. niger* isolates were able to produce fumonisin B2 in vitro. The analysis of ocharatoxin A and fumonisin B2 in artificially inoculated pomegranate fruits indicated that only a small percentage of the isolates were mycotoxin-producing isolates.

### 3.9. Citrus Fruit Rot

Aspergillus rot affects citrus fruits (*Citrus* spp.), including oranges (*C. sinensis*), lemons (*C. limon*), grapefruits (*C. paradisi*), and lime (*C. aurantiifolia*), and can occur in the field, postharvest, during storage and sale. The most common species associated with citrus fruit rot is *A. niger* followed by *A. flavus* (Table 8). However, in a study by Tournas and Katsoudas [159], *A. niger* was only recovered from lemons and not from other citrus fruit samples. Other species associated with Aspergillus rot in citrus include *A*. *westerdijkiae*, *A. aculeatus*, and *A. nidulans* (Table 8).

Ochratoxin and aflatoxin have been reported in citrus infected by *Aspergillus*, as well as production of mycotoxins by the fungi. In a study by Marino et al. [165], *A*. *westerdijkiae* inoculated on the surface of an orange fruit was able to produce ochratoxin A and caused visible rot lesions. The production of ochratoxin A increases at temperatures higher than 26 °C, which is the optimum temperature for mycotoxin production [168]. Aflatoxin was detected in orange samples with a high incidence of *A. flavus* [163]. *Aspergillus niger* from oranges collected from orchards in Mexico was found to produce aflatoxin B1 and fumonisin B1 [166].

### 3.10. Tropical Fruit Crops

Aspergillus rot in banana (*Musa* spp.), mango (*Mangifera indica*), papaya (*Carica papaya*), pineapple (*Ananas comosus*), and guava (*Psidium guajava*) is mainly associated with *A. niger* and *A. flavus.* Other species, such as *A. tamarii*, *A. fumigatus*, *A. terreus*, *A. ochraceous*, and *A. japonicus*, have also been reported to cause fruit rot in these tropical fruits (Table 9). Aspergillus rot has also been reported in jackfruit (*Artocarpus heterophyllus*) and sapota (*Manilkara zapota*) (Table 9). During harvest and handling, it is vital to minimize fruit bruising and wounding since during storage, bruised and wounded fruits are susceptible to *Aspergillus* infection [169].

Unlike grapes, fig, olives, and pomegranates, data on the contamination of tropical fruit crops with *Aspergillus* mycotoxins is currently lacking. This may be because *Aspergillus* infection of many tropical fruit crops is a secondary infection.

### 3.11. Strawberry Fruit Rot

Strawberry fruits (*Fragaria x ananassa*) are fleshy and soft, which makes them highly perishable and have a limited shelf-life [199]. These factors contribute to the susceptibility of strawberries to postharvest pathogens that cause fruit rot. Although *Botrytis cinerea* is the main postharvest pathogen of strawberry, causing gray mold, *Aspergillus* spp. have also been identified as pathogens, causing strawberry postharvest rot. *Aspergillus* species reported to be associated with strawberry rot include *A. niger*, *A. flavus*, *A. fumigatus*, *A. tubingensis*, *A. parasiticus*, and *A. terreus* (Table 10).

In a study by Palmer et al. [200], *A. tubingensis* was identified as a causal pathogen of strawberry rot in a field in California. However, the disease is of minor significance as the fungus was isolated during hot weather that favors the growth of *Aspergillus*. Most reports on strawberry rot caused by *Aspergillus* occur after harvest, particularly during storage and sale [201,202,203,204].

**Table 10:** *Aspergillus* spp. associated with strawberry fruit rot and fresh fruit.

Strawberry (*Fragaria x ananassa*)	*Aspergillus* spp.	Country	References
Fruit rot	*A. flavus*, *A. niger*	Qena city, Egypt	[203]
	*A. niger*, *A. fumigatus*	Lahore, Pakistan	[201]
	*A. tubingensis*	California, USA	[200]
	*A. terreus*, *Aspergillus* sp.	Indonesia	[204]
Fresh fruit and juice	*A. flavus*, *A. niger*, *A. parasiticus*	Saudi Arabia	[202]

Mycotoxigenic *A. flavus* and *A. parasiticus* associated with strawberry fruit rot were able to produce aflatoxins, as reported by Saleem [202] and Hussein et al. [203]. Saleem [202] reported that 30–60% isolates of *A. flavus* and *A. parasiticus* recovered from diseased fruits could produce aflatoxin B at varying concentrations. *Aspergillus niger* and *A. flavus* isolated from strawberry rot were also found to produce ochratoxin and aflatoxin, respectively [203]. These findings highlight the susceptibility of strawberries and strawberry products to contamination with aflatoxin and ochratoxin.

### 3.12. Apple Fruit Rot

Fruit rot in apples (*Malus domestica*) is caused by a range of postharvest pathogens, including *Aspergillus*. *Aspergillus* is not only associated with apple fruit rot; several species have also recovered from healthy apple fruits. The species isolated from apple fruit rot include *A. oryzae*, *A. flavus*, *A. niger*, *A. terreus*, and *A. versicolor* (Table 11).

*Aspergillus* infection often leads to the contamination of apple fruits with mycotoxins. Hasan [205] isolated *A. flavus* from 67% (from 100 samples) of rotted apples, with *A. flavus* being the most isolated fungus from healthy apples. Aflatoxins B1, B2, G1, and G2 were detected in the lesions of rotted apples. These findings demonstrate an association between *A. flavus* infection in apples and the occurrence of aflatoxins. *Aspergillus versicolor* isolated from rotten apples produced sterigmatocystin [209], which is a precursor of aflatoxin B1.

### 3.13. Peach, Cherry, and Kiwi Fruit Rot

Peach (*Prunus persica*), cherry (*Prunus avium*), and kiwi (*Actinidia deliciosa*) are also highly perishable and have short shelf-life, as well as being predisposed to *Aspergillus* infection (Table 12). In peaches, *A. flavus*, *A. niger*, and *A. aculeatus* are associated with peach rot [210,211,212]. Wounded peach fruits are more prone to infection by *Aspergillus* [213].

*Aspergillus* was the most dominant species recovered from postharvest sour cherries, and two species, *A. niger* and *A. penicillioides*, were identified [214]. *Aspergillus niger* was also reported as a causal pathogen of postharvest fruit rot in cherries in northern Greece [215].

Zhu et al. [216] isolated *A. flavus* from mature kiwifruit with brown lesions in southwestern Shaanxi, China, of which 15% of the fruits in the orchard exhibited soft rot symptoms. This study was the first to report *A. flavus* causing fruit rot in kiwis.

**Table 12:** *Aspergillus* spp. associated with fruit rot of cherry, peach, and kiwi.

Disease	*Aspergillus* spp.	Country	References
Cherry (*Prunus avium*)			
Postharvest fruit rot	*A. niger*	Imathia and Pella (northern Greece)	[215]
	*A. niger*, *A. penicilioides*	Lithuania	[214]
Peach (*Prunus persica*)			
Soft rot	*A. aculeatus*	Shaanxi, China	[212]
Fruit rot	*A. niger*	Gansu, China	[213]
	*A. flavus*	Imathia county, northern Greece	[210]
	*A. niger*	Jeddah, Saudi Arabia	[217]
Postharvest rot	*A. niger*	Rawalpindi, Pakistan	[211]
Kiwi (*Actinidia deliciosa*)			
Soft rot	*A. flavus*	southwestern Shaanxi, China	[216]

### 3.14. Tree Nuts

Common tree nuts are almonds (*Amygdalus communis* L.), Brazil nuts (*Bertholletia excelsa*), cashews (*Anacardium occidentale*), hazelnuts (*Corylus avellana*), pecans (*Carya illinoinensis*), pistachio nuts (*Pistacia vera*), macadamia (*Macadamia ternifolia*), and walnuts (*Juglans regia*) [218]. Among these, the most consumed tree nuts are almonds and walnuts, followed by pistachios, cashews, and hazelnuts [219].

*Aspergillus* infection of tree nuts occurs in the field, particularly in fruits wounded by insects as well as wounds caused during harvesting. Conidia are abundant as airborne inoculum, colonizing nuts and remaining present until their harvest, storage, and processing. These carry-over inoculums can remain in the produce until the processing and final product stages. When conditions favor fungal growth, the internal parts of nuts are often infected [220].

In a study on the occurrence of *Aspergillus* in pistachio, almond, and walnut, Bayman et al. [220] found that most common species of *Aspergillus* detected were *A. niger*, *A. flavus*, *A. nidulans*, *A. tamarii*, *A. ochraceus*, *A. melleus*, and *A. fumigatus*. Three species, *A. candidus*, *A. parasiticus*, and *A. terreus* were not common (less than 2% of the collected nuts). These results indicate that *Aspergillus* species are prevalent in tree nuts and suggest that the handling of nuts during harvest and postharvest has a major influence on the occurrence of mycoflora [220].

Several mycotoxigenic species have been reported, with aflatoxin and ochratoxin contamination also occurring in tree nuts. Tree nuts have a low sugar content, low moisture levels (particularly during storage and transportation), and high levels of water activity, which may contribute to the production of mycotoxins [221]. The majority of aflatoxin incidences have been reported in nuts damaged by insects or by the early splitting of the shell and hull [222]. However, mycotoxigenic *Aspergillus* species have also been found in nuts without insect damage or shell and hull splitting.

Aflatoxins have been detected at higher levels in several tree nuts, including almonds, Brazil nuts, pistachios, and walnuts [223,224,225]. According to Taniwaki et al. [226], the occurrence of aflatoxigenic *A. flavus* and other aflatoxigenic species on tree nuts is comparable to that on peanuts. The occurrence of ochratoxin A in almonds, hazel nuts, cashews, and walnuts was reported by Essawet et al. [227]. Although the contamination of tree nuts with ochratoxin A is often low, higher levels of mycotoxins have been detected occasionally [228].

### 3.15. Coffee Beans

Similar to other agricultural crops, coffee beans (Arabica and Robusta) are also infected by *Aspergillus* in the field and during storage, and are present at various production stages, including harvesting, postharvest, handling, processing, and transportation [229]. Black *Aspergillus* is the most commonly detected species in coffee beans. Black *Aspergillus* associated with coffee contamination include *A. carbonarius*, *A. niger*, *A. sclerotioniger*, *A. lacticoffeatus*, *A. sclerotiicarbonarius*, *A. aculeatinus*, *A. tubingensis*, and *A. foetidus*, among which some species are also ochratoxin A producers (Table 13). Other *Aspergillus* spp. recovered from coffee beans include *A. westerdijkiae*, *A. candidus*, *A. sydowii*, *A. ochraceus*, *A. parasiticus*, *A. fumigatus*, *A. flavus*, and *A. versicolor* (Table 13).

Infestation by the coffee berry borer (*Hypothenemus hampei*) has been found to increase the incidence of fungal contamination in coffee beans, as well as the levels of ochratoxin A [238]. Ochratoxin A produced during different stages of coffee processing reduces the quality of coffee and affects its taste [239]. Based on a study by Noonim et al. [231] on the production of ochratoxin by *Aspergillus* isolated from coffee beans in Thailand, *A. carbonarius*, *A. westerdijkiae*, and *A. steynii* were found to produce high amounts of ochratoxin A. An intermediate amount of ochratoxin A was produced by *A. niger* and *A. sclerotiorum*. *Aspergillus carbonarius* producing ochratoxin A with significant amount has been reported by Joosten et al. [240], Pardo et al. [241], and Leong et al. [232]. Although *A. niger* is among the most prevalent black *Aspergillus* contaminating coffee beans, the species is unlikely to be an important producer of ochratoxin A in coffee beans, as only a small percentage of *A. niger* isolates were able to produce the mycotoxin [230,231].

Different species of *Aspergillus* have been detected in coffee beans from coffee-producing countries. This suggests that the *Aspergillus* species depends on the climate of the geographic region, agricultural practices, pest infestation, and postharvest handling, including drying and storage [231,242]. In Brazil, *A. niger*, *A. ochraceus*, and *A. carbonarius* have been frequently isolated from coffee beans. Although *A. niger* was recovered at a higher percentage (63%), only 3% of the isolates produced ochratoxin A [230]. Three species, namely *A. niger*, *A. ochraceus*, and *A. carbonarius*, were also reported in coffee beans in Vietnam and Cameroon [232,235]. In Vietnam, Leong et al. [232] isolated *A*. *westerdijkiae* and *A. steyni*, but ochratoxin A was only produced by *A. carbonarius*., *A*. *westerdijkiae*, and *A. steyni* [232]. In the Phillipines, five species have been associated with the contamination of coffee, namely, *A. ochraceus*, *A. westerdijkiae*, *A. carbonarius*, *A. niger*, and *A. japonicus*, all of which were able to produce ochratoxin [234]. Four new species of black *Aspergillus*, *A. sclerotiorum*, *A. lacticoffeatus*, *A. sclerotiicarbonarius*, and *A. aculeatinus*, were identified in coffee beans in Thailand. Other species isolated from coffee beans included *A. niger*, *A. tubingensis*, *A. foetidus*, *A. carbonarius*, *A. niger*, and *A. westerdijkiae.* Among these, only *A. carbonarius* and *A. niger* were able to produce ochratoxins [237].

## 4. Other Plant Diseases Caused by *Aspergillus* spp.

Although *Aspergillus* species are typically weak or secondary pathogens, several species have been reported to cause foliar diseases, including leaf spot and leaf soft rot. Although *Aspergillus* are not common leaf spot pathogens, there are several reports that identify species of *Aspergillus* as leaf spot pathogens in several plants. Leaf spots appear as discolored spots or lesions on the leaf, with necrosis often occurring at the center of the lesion [243]. The spots on the leaf may coalesce and form irregular blight lesions. This disease usually occurs under conditions of continuous moisture and humidity. Most leaf spot pathogens including *Aspergillus* disseminate through conidia by rain splashing, irrigation, and wind dispersal [244].

*Aspergillus niger* has been reported to cause leaf spots in ginger (*Zingiber officinale*), in which severe infection caused defoliation [245] as well as leaf spot of avocado (*Persea americana*) [246]. *Aspergillus niger* has also been associated with soft rot of an ornamental plant, mother-in-law’s tongue (*Dracaena trifasciata*) [247], stem rot in lucky bamboo (*Dracaena sanderiana*) [248], and stem rot of *Adenium obesum* [246]. Another black *Aspergillus*, *A. tubingensis*, was reported to cause leaf spots on *Jatropha curcas* [249], *Helleborus* species [250], and cotton [251]. *Aspergillus tubingensis* has also been reported to cause black pods in tamarind (*Tamarindus indica*) [252], act as a pre-emergent pathogen of *Phoenix dactylifera* [253], and cause leaf rot in pak choi (*Brassica rape* spp. *chinensis*) [254]. Furthermore, three species, *A. niger*, *A. ustus*, and *A. flavus*, were identified as causal pathogens of foliar diseases in *Terminalia catappa*, a deciduous tropical tree [255]. *Aspergillus fumigatus* was also found to be a causal pathogen of marigold (*Tagetes erecta* and *T. patula*), causing foliage blight [256]. Recently, *A. versicolor* was identified as a pathogen causing severe fruit rot of tomato [257] and *A. niger* causing fruit rot of bilimbi (*Averrhoa bilimbi*) [258].

## 5. Control of *Aspergillus* Diseases

Integrated approaches are commonly employed to manage *Aspergillus* diseases both in the field and postharvest. *Aspergillus* infections in the field are often linked to wounds caused by insect infestations. The conidia, which reside in plant debris, soil, and mummified fruits, can be introduced into wounds through soil dust and rain splash. Therefore, maintaining sanitation in the field or orchard, which includes the removal of dead plant material and mummified fruits, is highly recommended [149].

Harvesting and postharvest activities predispose the crops and fruits to mechanical injury. Postharvest activities such as handling, sorting, grading, packing, and transportation require extensive operations, and often results in bruises, cracks, and cuts on the produce [259]. Minimizing injury during these activities reduces the risk of infection from fungal pathogens including *Aspergillus*.

Before packaging, individual fruits should be washed and cleaned in plenty of clean water to remove dirt and latex, as well as inoculum of pathogen that can cause rot disease during storage and transportation. Chlorine, chlorine dioxide, and hydrogen peroxide can serve as disinfectants for cleaning the fruits [260].

The use of fungicides is the main method of pre- and postharvest disease control. Fungicides should be applied during preharvest to prevent infection during postharvest storage and to control rot disease [261]. The use of fungicides to control grape berries infected with black *Aspergillus* and to reduce ochratoxin A levels was reviewed by Varga et al. [262]. Among the fungicides used were captan, fludioxonil, mepanipyrim, pyrimethanil, fluazinam, and iprodione mepanipyrim, pyrimethanil, fluazinam, and iprodione.

The potential of utilizing biological control methods for managing *Aspergillus* diseases has garnered significant interest. Among promising biocontrol agents tested is yeast to reduce infection, and mycotoxin production by different *Aspergillus* spp. *Saccharomyces cerevisiae* has the ability regulate production of aflatoxin by *A. flavus* during storage [263]. The growth of *A. carbonarius* can be inhibited by four yeast species, *Pichia kluyveri*, *Hanseniaspora uvarum*, *Meyerozyma guilliermondii*, and *Hanseniaspora clermontiae*, which is achieved through competition for available substrates [264].

Other potential methods to control *Aspergillus* growth and production of mycotoxin are utilizing essential oils and nanocoating. Essential oils extracted from thyme, cinnamon, basil, clove, mint, oregano, coriander, and anise have been reported to inhibit growth of *Aspergillus* [262]. Oregano and mint oils inhibited growth of *A. westerdijkiae* and ochratoxin production [265]. Nanocoating based on chitosan and propolis have been demonstrated to suppress growth of *A. flavus* and production of aflatoxins [266,267].

## 6. Conclusions

The compilation of different plant-pathogenic *Aspergillus* species along with the plant hosts demonstrated the genus/species global distribution. The plant pathogenic *Aspergillus* infection of a variety of crops may be due to a number of factors. Contributing factors might include the ability of *Aspergillus* species to inhabit agriculture environment, effective conidia dispersal by air, rapid adaptation to the host, growth and survival in a range of ecological conditions, and extensive use of chemicals in agricultural practices. However, there are still many scientific problems and knowledge gaps that need to be addressed, including the adaptation to various ecological areas, host-switching, and infection-causing mechanisms in various crops and plants.

*Aspergillus* is currently regarded as a potential emergent plant pathogen and probably will lead to future outbreaks of plant disease. In this situation, if plant pathogenic *Aspergillus* species that cause serious diseases are not detected and identified in a timely manner, and appropriate plant disease management approaches are not implemented, food safety could be adversely affected, which would have a significant economic impact.

## Figures and Tables

**Table 1:** *Aspergillus* species associated with diseases of peanuts.

Peanuts (*Arachis hypogaea*)	*Aspergillus* spp.	Country	References
Diseases			
Crown rot/collar rot	*A. niger*	Oklahoma, USA; Andhra Pradesh, Karnataka and Tamil Nadu, India; Jackson County, Florida, USA.	[41,42,43,44,45]
Yellow mold	*A. flavus*, *A. parasiticus*	Tropical and subtropical areas (country not stated)	[46,47,48,49,50]
Root rot	*A. niger*	Laizi District, Shandong Province, China	[51]

**Table 2:** *Aspergillus* species associated with cotton boll rot.

Cotton (*Gossypium herbaceum*)	*Aspergillus* spp.	Country	References
Cotton boll rot	*A. niger*	Oklahoma, USA	[52]
	*A. flavus*, *A. niger*	California, USA; Bangladesh	[53,54]
	*A. flavus*	Southeast and Mid-South states USA	[55,56,57,58]

**Table 3:** *Aspergillus* species associated with black mold of onion and garlic.

Onion (*Allium cepa* L.) and Garlic (*Allium sativum* L.)	*Aspergillus* spp.	Country	References
Black mold	*A. niger* (onion)	Worldwide	[59]
	*A. niger*, *A. ochraceus* (garlic)	USA, China	[60]
	*A. awamori* (garlic)	Korea	[61]
	*A. welwitschiae* (onion)	Taif region, Saudi Arabia; Stara Pazova, Serbia; Paraná State, Brazil.	[62,63,64]
	*A. niger* (onion)	Shambat, Sudan; Wellesbourne, UK	[65]
	*A. awamori* (onion)	Hungary	[66]

**Table 5:** *Aspergillus* spp. associated with fig fruit rot and dried fig.

Fig (*Ficus carica*)	*Aspergillu*s spp.	Country	References
Fruit rot/ Aspergillus rot	*A.cabonarius*, *A. japonicus*, *A. niger*	California, USA	[117]
	*A. flavus*	California, USA	[118]
Dried fig	*A. flavus*, *A. parasiticus*	Brazil	[119,120,121,122]

**Table 6:** *Aspergillus* spp. associated with olive fruit rot and healthy fruits.

Olive (*Olea europaea*)	*Aspergillus* spp.	Country	References
Fruit rot	*A. ochraceus*	Tarom-Zanjan Province, Tabriz, Iran	[134]
	*A. fumigatus*	Halkidiki, Kalamata, Athens	[135]
	*A. niger*	Karak, Jordan	[136]
	*A. flavus*	Gharb and Zoumi, Morocco	[133]
	*A. niger*, *A. fumigatus*	Sidi Kacem, Meknes, Fes, Taounate, Sefrou, Khenifra, Errachidia, Goulmima, and Marrakech, Morocco	[130]
Healthy fruits	*A. niger*, *A. tubingensis*	Canakkale province, Turkey	[136]

**Table 7:** *Aspergillus* spp. associated with pomegranate fruit rot.

Pomegranate (*Punica granatum*)	*Aspergillus* spp.	Country	References
Disease			
Heart rot	*A. niger*	Cairo, Egypt	[154]
Fruit rot	*A. niger*	California, USA	[149]
	*A. tubingensis*	China	[155]
Soft rot and dry rot	*A. niger*	Shaanxi, China	[153]
Postharvest rot	*A. tubingensis*, *A. welwitschiae*, *A. uvarum*, *A. japonicus*	Southern Italy	[156,157]

**Table 8:** *Aspergillus* spp. associated with citrus fruit rot.

Citrus (*Citrus* spp.)	*Aspergillus* spp.	Country	References
Lemon	*A. niger*	Washington D.C., USA	[159]
Lemon and grapefruit	*A. niger*	Islamabad, Rawalpindi, Taxila, and Wah districts Pakistan	[160]
Lemon, sweet lemon, lime, sweet orange	*A. niger*, *A. flavus*	Adamawa state, Nigeria	[161]
Lemon	*A. flavus*	Erzurum, Turkey	[162]
Orange	*A. flavus*, *A. niger*	Oyo State, Nigeria	[163]
	*A. niger*	El-beida, Libya	[164]
	*A. westerdijkiae*	Italy	[165]
	*A. niger*, *A. aculeatus*, *A. nidulans*	Mexico	[166]
	*A. niger*	Nigeria	[167]

**Table 9:** *Aspergillus* spp. associated with diseases of tropical fruit crops.

Fruit Crop/Disease	*Aspergillus* spp.	Country	References
Banana (*Musa* spp.)			
Fruit rot	*A. niger*, *A. flavus*	Dhaka, Bangladesh	[170]
	*Aspergillus* spp.	South Gujarat	[171]
	*A. niger*, *A. flavus*	Kono, Nigeria	[172]
	*A. niger*, *A. fumigatus*, *A. flavus*	Sokoto, Nigeria	[173]
	*A. tamarii*	Malaysia	[174]
Crown rot	*A. niger*, *A. flavus*	Jimma town, Ethiophia	[175]
	*Aspergillus* sp.	Kerala, India	[176]
Mango (*Mangifera indica*)			
Fruit rot	*A. niger*	Sri Lanka, Iran	[177,178]
	*A. flavus*, *A. niger*	Saudi Arabia, Faisalabad, Pakistan	[179,180]
	*A. niger*, *A. oryzae*	Nasarawa State, Nigeria	[181]
	*A.niger*, *A. flavus*, *A. fumigatus*, *A. terreus*	Dhaka, Bangladesh	[170]
Pineapple (*Ananas comosus*)			
Fruit rot	*A. flavus*	Nigeria	[167]
	*A. flavus. A. niger*	Osun State, Nigeria	[182]
	black *Aspergillus*	Anambra State, Nigeria	[183]
Papaya (*Carica papaya*)			
Fruit rot	*A. niger*, *A. terreus*, *A. flavus*, *A. ochraceous*, *A. tamarii*, *A. fumigatus*	Gorakhpur, India	[184]
	*A. flavus*	Maharashtra, India	[185]
	*A. niger*	Uttar Pradesh, India	[186]
	*A. niger*, *A. flavus*	Osun State, Nigeria	[182]
Guava (*Psidium guajava*)			
Crown rot	*A. flavus*, *A. fumigatus*, *A. japonicus*, *A. niger*, *A. tamarii*	Nueva Ecija, Phillippines	[187]
Fruit rot	*A. awamori*	Lahore, Pakistan	[188]
Soft rot	*A. niger* var. *awamori*	India, Malaysia	[189,190,191]
Dry rot	*A. fumigatus*	Nigeria	[192]
	*A. niger*	Ethiopia	[193]
	*A. niger*, *A. flavus*, *A. parasiticus*	Beheira, El-Sharkia and Qualubia governorates, Egypt	[194]
	*A. niger*, *A. awamori*	Aurangabad, India	[195]
Jackfruit (*Artocarpus heterophyllus*)			
Fruit rot	*A. niger*	Nayarit, Mexico	[196]
Sapota (*Manilkara zapota*)			
Fruit rot	*A. minisclerotigenes*	Gujarat, India	[197]
	*A. niger*	Maharashtra, India	[198]

**Table 11:** *Aspergillus* spp. associated with apple fruit rot.

Apple (*Malus domestica*)	*Aspergillus* spp.	Country	References
Fruit rot	*A. flavus*, *A. niger*	Assuit, Egypt	[205]
	*A. oryzae*	Riyadh, Saudi	[206]
	*A. flavus*, *A. niger*, *A. terreus*	Babylon, Iraq	[207]
	*A. niger*, *A. terreus*	Lagos State, Nigeria	[208]
	*A. versicolor*	Slovak Republic	[209]

**Table 13:** *Aspergillus* spp. associated with coffee cherry and coffee bean reported in several countries.

Coffee (*Coffea* spp.)	*Aspergillus* spp.	Country	References
*Coffea arabica*—cherries and beans	*A. ochraceus* (and possibly related species), *A. carbonarius*, *A. niger*	Alta Paulista, Sorocabana, Alta Mogiana, and Cerrado Mineiro, Brazil	[230]
*Coffea arabica* and *Coffea canephora* var. robusta	*A. melleus*, *A. sclerotiorum*, *A. steynii*, *A. westerdijkiae*, *A. aculeatinus*, *A. foetidus*, *A. niger*, *A. tubingensis*	Chiang Mai, Chumphon, Thailand	[231]
Green coffee bean (Robusta and Arabica)	*A. carbonarius*, *A. niger*, *A. ochraceus* and related species in section Circumdati	southern and central Vietnam	[232]
Coffee bean	*A. carbonarius*, *A. niger*, *A. ochraceus*	Paraná, São Paulo and Minas Gerais, Brazil	[233]
*Coffea arabica*, *Coffea canephora* var. Robusta, *Coffea liberica*, *Coffea excelse*a	*A. ochraceus*, *A. westerdijkiae*, *A. carbonarius*, *A. niger*, *A. japonicus*	Benguet, Ifugao; Abra, Cavite, Ifugao, Cavite, Philippines	[234]
Dry parchment, dry cherries and green coffee beans	*A. carbonarius*, *A. niger*, *A. ochraceus*	west region of Bafoussam and Dschang, Cameroon	[235]
*Coffea arabica*, *Coffea canephora* L. var. *robusta* (Robusta coffee) green coffee beans	*A. candidus*, *A. sydowii*, *A. niger*, *A. ochraceus*, *A. parasiticus*, *A. fumigatus*, *A. flavus*, *A. versicolor*	Brazil, Timor, Honduras, Angola, Vietnam, Costa Rica, Colombia, Guatemala, Nicaragua, India, and Uganda	[236]
Arabica—parchment and green coffee beans	*A. niger A. tubingensis*, *A. foetidus*	North Thailand	[237]
Robusta—dried coffee cherries and green coffee beans	*A. carbonarius*, *A. niger*, *A. westerdijkiae*, *A. aculeatinus*, *A. sclerotiicarbonarius*	South Thailand	[237]
Coffee beans	*A. brasiliensis*, *A. flavus*, *A. lanosus*, *A. niger*, *A. ochraceus A. oryzae*, *A. ostianus*, *A. sulphureus*, *A. tamarii*, *A. tubingensis*	Minas Gerais, Brazil	[238]
